# Advances in Nanoparticle Drug Delivery Systems for Anti-Hepatitis B Virus Therapy: A Narrative Review

**DOI:** 10.3390/ijms222011227

**Published:** 2021-10-18

**Authors:** Jing Miao, Peng Gao, Qian Li, Kaifeng He, Liwen Zhang, Junyan Wang, Lingfei Huang

**Affiliations:** 1 Department of Pharmacy, The Children’s Hospital, Zhejiang University School of Medicine, National Clinical Research Center for Child Health, Hangzhou 310052, China; miaojing@zju.edu.cn (J.M.); gaopeng@zju.edu.cn (P.G.); kyllfanhe@yeah.net (K.H.); 6512070@zju.edu.cn (L.Z.); 2 Zhejiang Provincial Key Laboratory for Drug Evaluation and Clinical Research, Hangzhou 310003, China; 3 Department of Pharmacy, The First Affiliated Hospital, Zhejiang University School of Medicine, Hangzhou 310003, China; 1310024@zju.edu.cn

**Keywords:** hepatitis B virus, nanoparticle, drug delivery, anti-HBV therapy

## Abstract

Chronic hepatitis B (CHB) is an infectious viral disease that is prevalent worldwide. Traditional nucleoside analogues, as well as the novel drug targets against hepatitis B virus (HBV), are associated with certain critical factors that influence the curative effect, such as biological stability and safety, effective drug delivery, and controlled release. Nanoparticle drug delivery systems have significant advantages and have provided a basis for the development of anti-HBV strategies. In this review, we aim to review the advances in nanoparticle drug delivery systems for anti-hepatitis B virus therapy by summarizing the relevant literature. First, we focus on the characteristics of nanoparticle drug delivery systems for anti-HBV therapy. Second, we discuss the nanoparticle delivery systems for anti-HBV nucleoside drugs, gene-based drugs, and vaccines. Lastly, we provide an overview of the prospects for nanoparticle-based anti-HBV agents.

## 1. Introduction

Hepatitis B is an infectious disease caused by the hepatitis B virus (HBV) and is prevalent worldwide [1]. HBV belongs to the family of hepatophilic DNA viruses, which can cause acute and chronic hepatitis. Patients with chronic infection are generally asymptomatic; however, HBV carriers are at a high risk of developing cirrhosis or liver fibrosis, as well as liver cancer [2]. Currently, the clinical treatment for chronic hepatitis B (CHB) infection is limited to interferon (IFN) therapy and nucleoside analogues (NAs) [3]. IFN therapy is associated with significant adverse reactions, poor human tolerance, and low virus negative conversion rate; therefore, only a small number of patients are candidates for the therapy [4]. NAs, especially the first-line drugs, entecavir and telbivudine, significantly inhibit HBV replication and are well tolerated by the human body [5]. However, all NAs are associated with long treatment cycles, a high rebound rate after drug withdrawal, and a high rate of clinical resistance [6].

The HBV genome is a partially double-stranded relaxed cyclic DNA molecule, encapsulated by the core antigen (HBcAg) and an envelope containing the surface antigen (HBsAg) [7]. HBV enters the host cell via the sodium taurocholate co-transporting polypeptide pathway, removes the envelope protein, releases core particles, and forms a relaxed circular DNA (rcDNA) in the nucleus of the host cell. A partially double-stranded DNA of the rcDNA is assembled by reverse transcription to form a highly stable micro-chromosome structure; namely, covalently closed circular DNA (cccDNA). The cccDNA can be transcribed into all viral messenger RNAs (mRNAs) including pre-genomic RNA (pgRNA) as a template for the replication of offspring viruses. The pgRNA can encode viral capsid and polymerase proteins and form rcDNA through reverse transcription by being packaged in a nuclear capsid. Some of the mature rcDNA is packaged in the HBsAg protein to form progeny viruses, which are released extracellularly and infect other hepatocytes. Another portion of the rcDNA returns to the nucleus and converts into cccDNA using the host cell DNA repair response, which complements the cccDNA reservoirs in the nucleus and forms a “continuous stream” of viral replication template reservoirs [8,9].

**Figure 1:**
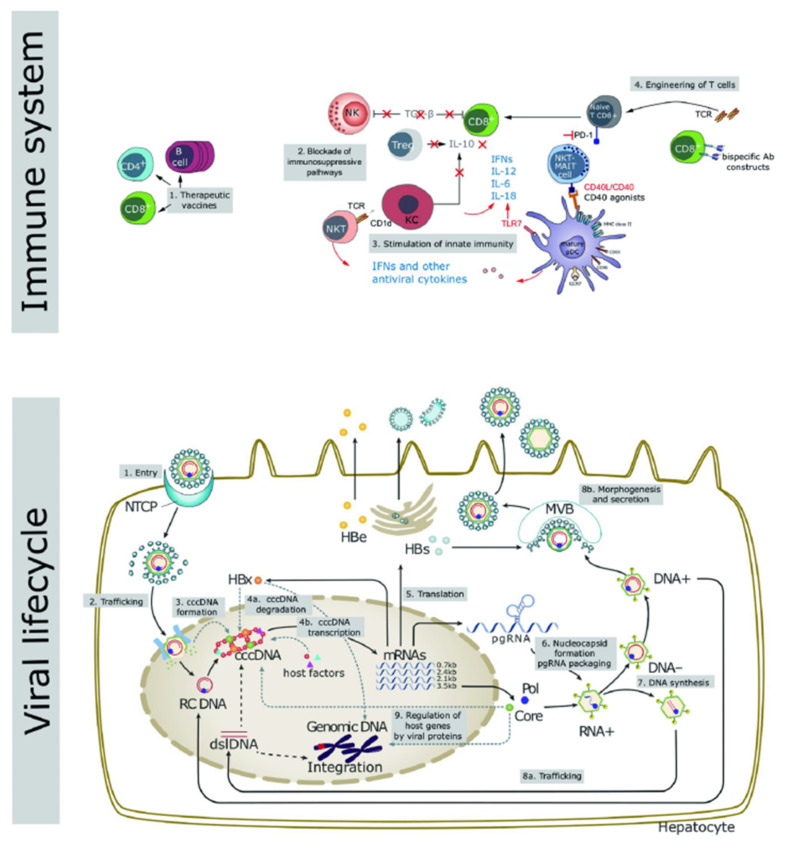
Key aspects of the viral life cycle and host immune system representing the novel targets for the development of innovative therapeutic strategies to counteract HBV infection. Reproduced from Liver International 2017, 37 (Suppl. 1), 33–39.

The inability of existing drugs to enter the nucleus and clear the cccDNA is the main cause of CHB infection and recurrence in “functional cure” patients with negative HBsAg findings after drug withdrawal [10,11,12]. In addition, integration of the HBV and host cell genomes occurs during the process of viral replication, which is closely related to chronic infection and the occurrence of hepatoma [13,14]. In recent years, several studies have been performed to research and analyze the life cycle of HBV, through which many novel promising drug targets have been unveiled (Figure 1) [15].

Host immunity includes innate and adaptive immunity, and the majority of the patients with normal immune function can clear the HBV DNA and cccDNA during acute infection, due to which the disease becomes self-limiting. However, CHB infection is associated with a dysfunctional immune response. As shown in Figure 1, therapeutic targets based on host immunity include therapeutic vaccines, blocking the immunosuppressive pathways, activation of innate immunity, and CAR-T therapy.

Research on novel drugs and pharmaceutical preparations provides new strategies to determine the treatment, design effective drug combinations, address clinical issues such as long-term control of chronic infection, and achieve functional cure of hepatitis B.

In recent years, many novel anti-hepatitis B drugs were developed on the basis of the life cycle of the virus and host immune mechanisms [16,17,18]. Despite the promising effects of the novel agents, there are certain critical factors that influence the curative effect, such as biological stability and safety, effective drug delivery, and controlled release. Several researchers have developed nanoparticle delivery systems for traditional NAs, which can reduce dosage and toxicity and improve the therapeutic index [19,20,21,22]. Regarding the novel anti-hepatitis B drug targets, different nanoparticle drug-delivery systems have played an indispensable role in improving the antiviral effect and reducing the development of resistant strains through targeted drug delivery, thus blocking the evolution of the disease.

**Figure 2:**
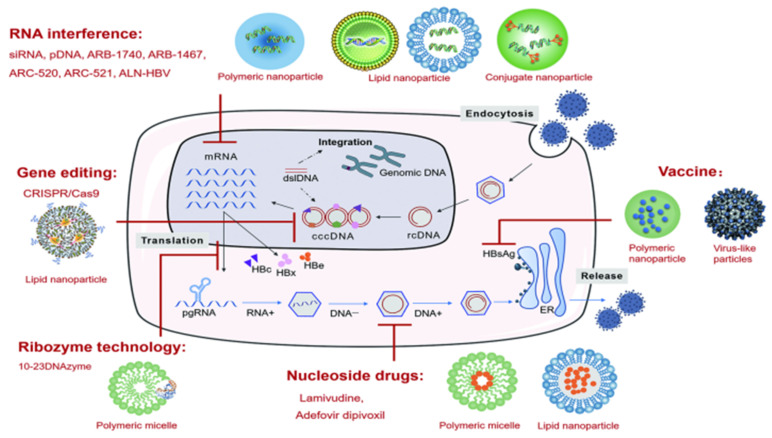
Types of nanoparticle delivery systems for anti-HBV therapy.

Up to now, there has been a dramatic increase in various nanoparticle formulations for anti-HBV therapy (Figure 2), and an overview of the current literature is needed. Therefore, this review aims to summarize the characteristics of nanoparticle delivery systems for various anti-HBV therapeutic strategies, including nucleoside drugs, gene therapy, and vaccines. The advantages of nanoparticle delivery systems are introduced, and the latest progress in enhancing targeted drug delivery in anti-HBV therapy is discussed. Lastly, possible development directions are predicted.

## 2. Methods

For the purposes of this review, a bibliographic search was carried out in PubMed using the following search string: [(nanoparticles or micelles) and (Hepatitis B or HBV)], resulting in 553 articles. Then, we manually screened the obtained articles with a specific focus on nucleoside drugs, ribozyme, RNA interference, gene editing, and vaccine. Finally, 24 papers were analyzed because they were directly related to the nanoparticle delivery systems for anti-HBV nucleoside drugs (cited in Table 1), anti-HBV genetic drugs (cited in Table 2), and anti-HBV vaccines (cited in Table 3).

## 3. Characteristics of Nanoparticle Drug Delivery Systems for Anti-HBV Therapy

Nanoparticle drug delivery systems refer to drug preparations with particle sizes ranging from 1 nm to 1000 nm. The particle size, potential, morphology, and internal structure of the nanoparticle drug delivery system are controllable. Several nanoparticle drug delivery systems with specific biochemical characteristics have been designed to achieve personalized and targeted treatment. Nanoparticle drug delivery systems have significant advantages in the treatment of hepatitis B infection. These include the ability to (1) passively target the liver tissue through hepatic macrophages (mainly Kupffer cells), (2) be modified with hepatocyte-targeting molecules to achieve active distribution of the drug(s) in the liver, (3) achieve environment-responsive drug release by bonding environment-sensitive ligands based on the intracellular environment of liver cells, and (4) encapsulate a variety of drug molecules simultaneously, thereby achieving codelivery of multiple therapeutic drugs with different efficacies.

**Figure 3:**
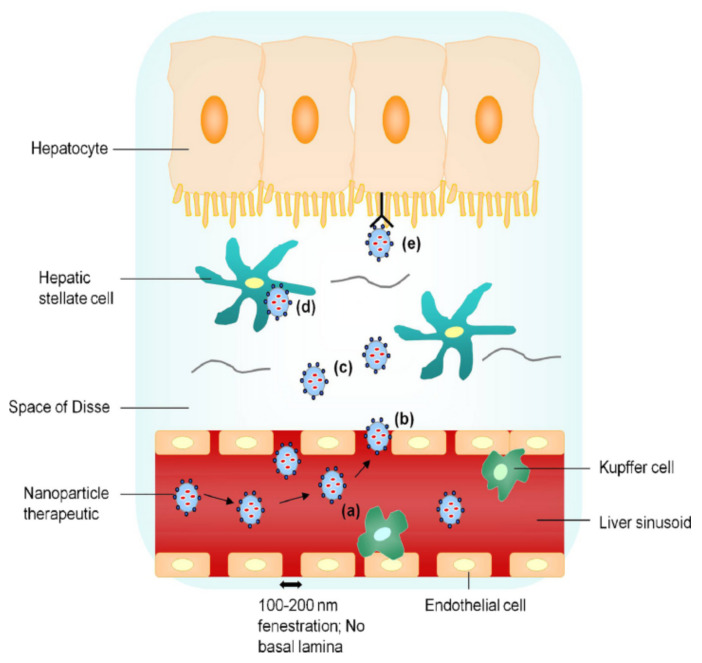
Liver-targeting process of nanoparticle drug delivery system. Reproduced from Nanotoday 2010, 5(4), 296–312.

As HBV mainly infects hepatocytes [23], targeting both liver tissue and hepatic parenchymal cells is essential to improve the therapeutic efficacy of anti-hepatitis B drugs. As shown in Figure 3 [24], the uptake processes of nanoparticle drug delivery systems are as follows: (a) prevention of ingestion by Kupffer cells (by use of nanoparticles <200 nm), (b) passage of nanoparticles <200 nm through endothelial gaps, (c) diffusion of the nanoparticles across the loosely organized extracellular matrix in the Disse space, (d) nonspecific endocytosis of hepatic stellate cells, and (e) receptor-mediated active uptake of hepatocytes.

However, the in vivo uptake of nanoparticle delivery systems by hepatocytes is limited. Targeting hepatocytes specifically was shown to reduce the accumulation of vectors in hepatic sinusoidal endothelial cells, as well as the hepatotoxicity [25]. Therefore, hepatocyte targeting is significant for the safe and efficient application of nanoparticle delivery systems. Currently, the main strategies include the following:

(a) Controlling the particle size below 200 nm, which reduces phagocytosis by nonparenchymal cells such as Kupffer cells, and further controlling the particle size at 50–100 nm, which is conducive to uptake by the hepatocytes.
(b) Modification of the nanoparticle delivery system to achieve targeted uptake by hepatocytes [26].

The surface receptors of hepatocytes include asialoglycoprotein receptor (ASGPR) [27], glycyrrhizic acid [28], mannose [29], and high-density lipoprotein receptors [30]. Among them, the ASGPR is a highly efficient endocytosis receptor specific to hepatocytes and is used widely. Human ASGPR is mainly expressed in hepatocytes, and there are approximately 500,000–1,000,000 receptors per person. The reported ASGPR-specific ligands include galactose [31], lactose [32], acetylgalactosamine [33], and asialofetuin [34]. Ligand modification could promote the delivery of active targeted nanocarriers to the hepatocytes.

## 4. Nanoparticle Delivery Systems for Anti-HBV Nucleoside Drugs

Anti-HBV nucleoside drugs, especially lamivudine and adefovir dipivoxil, are associated with long treatment cycles, a high rebound rate after drug withdrawal, and a high rate of drug resistance. Studies on nanoparticle delivery systems for nucleoside drugs, including polymeric micelles and lipid nanoparticles, have attracted wide attention to improve their efficacy and reduce the incidence of drug resistance (Table 1).

### 4.1. Polymeric Micelles

In most cases, hydrophobic drugs are incorporated into the hydrophobic core of polymeric micelles via hydrophobic interactions and other interactions, such as ligand coordination bonding [35,36] and electrostatic interaction [37,38,39,40]. Thus, undoubtedly, the tenuous hydrophobicity of nucleoside drugs should be adjusted. Li et al. [20] developed a chitosan stearate conjugated micellar delivery system (CSO-SA/LAS) loaded with lamivudine stearate (LAS) and investigated its antiviral activity in vitro. When the grafting rate was 3.79%, the entrapment efficiency of CSO-SA was 99.48% and the drug loading capacity was 39.04%. Compared to free lamivudine, CSO-SA/LAS significantly inhibited the expression of HBsAg and hepatitis B e antigen (HBeAg) and had a higher inhibition rate on HBV-DNA replication, which significantly improved the antiviral effect of lamivudine. Some studies further utilized specific ligands to form polymeric nanoparticles for liver targeting. Mishra et al. [21] developed glycyrrhizin (GL)-modified low-molecular-weight chitosan nanoparticles (GL-CS-NPs) to encapsulate lamivudine. The study reported that the release of lamivudine from nanoparticles showed a biphasic pattern, with an initial burst and subsequent sustained release. Compared to the lamivudine solution, GL-CS-NPs demonstrated better liver targeting ability and decreased tissue damage.

### 4.2. Lipid Nanoparticles

Lipid nanoparticles generally consist of a matrix of physiological or physiologically related lipids and exhibit more advantages, such as improved kinetic stability, drug solubility, and controlled drug release when compared with other drug delivery systems [41]. Therefore, lipid nanoparticles assembled with anti-HBV nucleoside drugs can improve their antiviral efficacy. Zhang et al. [19] developed adefovir dipivoxil solid lipid nanoparticles (SLNs) via the solvent diffusion method and investigated the in vitro anti-HBV efficacy. Compared to the free drug, SLN-loaded adefovir dipivoxil significantly inhibited the expression of HBsAg and HBeAg, as well as HBV DNA replication. Furthermore, multiple lipid nanoparticles (MLNs) are candidates to become the basis of novel drug delivery systems due to their smaller size and long-term storage stability [42]. Cavalcanti et al. [22] developed MLNs via hot homogenization combined with high shear and ultrasound and compared the system with nanostructured lipid carriers (NLCs). The results showed that MLNs had a higher drug loading capacity and particle stability than NLCs, and they were suitable for oral administration.

## 5. Nanoparticle Delivery Systems for Anti-HBV Gene Therapy

In recent years, novel anti-HBV gene-based drugs have become the hotspot of therapeutic research [43,44,45]. Currently, the strategies for anti-HBV gene therapy mainly include ribozyme, RNA interference (RNAi), and gene editing technologies. However, several parameters affect the efficacy of anti-HBV gene-based drugs in vivo. For example, these drugs are easily degraded by enzymes in the blood and liver and are hydrophilic macromolecules with a negative charge, which do not efficiently penetrate the cell membrane. Therefore, effective vectors are required for the therapeutic use of gene-based drugs. Viral vectors demonstrate high transfection efficiency; however, viral proteins can induce an immune response, and there are potential safety problems such as virus replication in vivo [46]. The majority of the clinical trials on gene therapy with viral vectors remain in clinical phase I (safety trial phase). Therefore, nonviral gene vectors have attracted extensive attention and have been applied in the research of various anti-HBV gene-based drugs (Table 2).

### 5.1. Ribozyme Technology

A ribozyme is a type of RNA or DNA with the catalytic activity that can specifically cut RNA fragments and block expression, making it attractive as a therapeutic tool for the inactivation of both viral RNA and mRNA associated with various diseases [61].

Xia et al. [56] designed an RNase targeting preS1 antigen and the surface region of HBV pgRNA. The inhibition rate of HBV-related gene expression was up to 95% in a mouse model. A DNAzyme is a specifically structured DNA sequence with catalytic RNA-cleaving activity, and 10–23 DNAzyme combines specifically with target mRNA, thereby blocking the expression of the corresponding mRNA [47]. Hou et al. [62,63] designed 10–23 DNAzyme fragments of different lengths to complement the HBV X protein and S and C genes. In vitro experiments showed that the designed ribozyme could significantly inhibit the expression of HBx protein, as well as HBsAg and HBeAg, and effectively inhibit the replication of HBV. In order to achieve more precise capture by the hepatic cell and reduce side effects, environment-sensitive strategies can be adopted to acquire stimulus-responsive drug complexes. Miao et al. [47] designed micelles loaded with 10–23 DNAzyme for the HBV S and C genes. They initially delivered the 10–23 DNAzyme via stearic acid-grafted chitosan oligosaccharide (CSSA). Compared to the commercial liposome delivery system, CSSA had the advantages of low toxicity, good targeting, and good anti-HBV efficacy. In the subsequent study [48], CSSA was further treated with a disulfide bond, and improved chitosan-SS-Octadecylamine (CSSO) micelles were obtained. In response to the high glutathione reduction environment in the parenchymal cells of the liver, the micelles could rapidly break the disulfide bond in the target region, release the drug, and improve the efficacy.

### 5.2. RNA Interference Technology

RNAi is a process that involves highly conserved, short, double-stranded RNAs that induce the degradation of homologous RNA, resulting in post-transcriptional silencing of specific genes. RNAi drugs can target the pgRNA of HBV and effectively inhibit the protein produced by the cccDNA and integrated virus DNA. RNAi technology has the potential for a broad range of applications in hepatitis B gene therapy [64,65]. Currently, there are three main types of nanoparticle drug delivery systems based on RNAi, namely, lipid [66], polymeric [67], and conjugate nanoparticles [68].

#### 5.2.1. Lipid Nanoparticles (LNPs)

Among the nanoparticle drug delivery systems based on RNAi above, LNPs represent one of the most widely studied delivery systems for RNAi [69]. RNAi lipid-based nanocarriers are able to provide protection from serum nucleases and extend circulation, which results in a higher access to the target tissue. Carmona et al. [49] developed a polyethylene glycol (PEGylated) small interfering RNA (siRNA) lipid carrier system. The anti-HBV preparation was manufactured by condensation of the siRNA (component A) and cationic liposome (component B) to form AB nuclear particles. PEGylated siRNA nanoparticles (also known as siRNA ABC nanoparticles) with a diameter of 80–100 nm were obtained by coupling PEG (component C) on the lipid surface. The inhibition rate of viral replication was three times higher than that observed in the control group after repeated and systematic administration of siRNA ABC nanoparticles to HBV transgenic mice.

Tekmira Pharmaceuticals Corporation/Arbutus Biopharma has developed three therapeutic targets using the RNAi technology for the HBV genome to degrade all viral mRNA transcription templates [70]. The three unique and highly conserved targets influence the coding expression of all genotypes of the HBV antigen and could be an effective “omnipotent” drug for all strains from genotype A to H in patients with CHB. Thus, the threshold of viral resistance can be increased significantly, and the resistance to other anti-HBV drugs can be reduced simultaneously. The LNP developed by Tekmira Pharmaceuticals Corporation/Arbutus Biopharma is currently the most widely accepted RNAi transport technology. The LNP components of the two products under research are different, wherein one includes the third-generation nanoparticle, whereas the other adopts the new fourth-generation LNP. TKM-HBV can reduce the expression of HBV DNA, cccDNA, HBsAg, HBeAg, and HBcAg in the liver and serum, as well as reduce the reactivation of HBV and cccDNA by activating humoral and T-cell immune mechanisms.

ARB-1740 [50] is a novel RNAi drug that has entered the clinical research stage. Three types of siRNA targets at three highly conserved regions of the HBV genome are simultaneously packaged and delivered using the LNP technology. The LNP component helps protect the siRNA from degradation by plasma nucleases. After being internalized by hepatocytes, it can fuse with the endoplasmic membrane in a pH-dependent manner and release the encapsulated siRNA into the cytoplasm. An animal study on cells infected with HBV demonstrated that ARB-1740 inhibited the expression of HBsAg, HBeAg, and HBcAg proteins, as well as HBV DNA [51]. ARB-1740 showed genotype activity in vitro and in vivo, which effectively inhibited the replication of NA-resistant HBV variants. ARB-1740 combined with capsid inhibitor and PEGylated IFN-α was found to significantly reduce the expression of HBsAg in the liver, which was related to the induction of an effective innate immune response in a human chimeric mice HBV model.

Moreover, the RNAi candidate, ARB-1467 (Arbutus Biopharma), contains three triggers for all four HBV transcripts and has been shown to reduce all viral antigen levels, cccDNA, and HBV DNA in preclinical studies. ARB-1467 uses a clinically proven delivery technology, the Arbutus proprietary LNP platform, which has been tested in hundreds of patients. In phase 2 clinical trials, the researchers found that a single treatment significantly reduced serum HBsAg levels, while multiple treatments had an additional effect [52].

#### 5.2.2. Polymeric Nanoparticles

Polymeric carriers have been extensively applied in siRNA-mediated therapy, and impressive progress has been made [71]. Furthermore, nanoparticles formulated from polymers have also shown potential for sustained nucleic acid (e.g., pDNA and siRNA) delivery [72].

Zeng et al. [53] used a biodegradable cationic polymer, polylactide glycolide (PLGA)-grafted chitosan (CHS), to develop PLGA-CHS nanoparticles (PLGA-CHS-NS) with a diameter of approximately 60 nm via the spontaneous emulsion diffusion method to deliver plasmid DNA. Adding CHS to the PLGA matrix improved the loading efficiency and cell absorption ability of PLGA nanoparticles. HepG2.2.15 was used as the viral cell model and was co-incubated with the nanoparticles. The results showed that the uptake of PLGA-CHS-NS was significantly stronger than that of PLGA-NS. In addition, the HBV gene silencing efficiency of PLGA-CHS-NS was significantly higher than that of PLGA-NS and the plasmid DNA-free solution.

The siRNA anti-HBV drugs, ARC-520 [54] and ARC-521 [55], developed by Arrowhead Pharmaceuticals (United States) can block the expression of some HBV proteins at the mRNA level, and they are expected to become the novel “functional cure” drugs. ARC-520 intervenes at the mRNA level, upstream of the HBV reverse transcription process, and is the target of nucleoside and NAs, which are most commonly used in clinical practice. ARC-520 and ARC-521 adopt the unique “dynamic polyconjugate” polymeric nanoparticle delivery system of Arrowhead Pharmaceuticals. The principle is that RNAi can block the expression of some HBV proteins, inhibit proliferation of the virus, and stimulate the human immune system to remove the remaining virus, to achieve an “immune clean state”, characterized by seronegativity of HBsAg with or without seroconversion. Unfortunately, the results of recent primate toxicology studies revealed that ARC-520 was associated with death following high doses and other safety problems. Nevertheless, in order to maximize the therapeutic efficacy of siRNA and reduce the risk for adverse effects, rational optimization is eagerly needed. Therefore, Arrowhead announced discontinuation of the research and development of both ARC-520 and ARC-521 in 2018. However, individual studies are in progress. Yuen et al. [73] evaluated the degree of HBsAg decline in response to multiple doses of ARC-520 compared to placebo in two randomized, multicenter studies in nucleoside/nucleotide analogue reverse transcriptase inhibitor (NUC)-experienced patients with HBeAg-negative (E-neg) or positive (E-pos) disease. The results revealed that ARC-520 was active in both E-neg and E-pos NUC-experienced HBV patients.

#### 5.2.3. Conjugate Nanoparticles

ALN-HBV, developed by Alnylam Pharmaceuticals (Cambridge, CA, USA), uses *N*-acetylgalactosamine (GalNAc) siRNA conjugates, which enhances the chemical stability of galactosamine [56]. The results of their clinical research revealed that the molecule has strong HBV target gene knockout ability and long-lasting efficacy. The proposed treatment regimen is subcutaneous administration once a month, quarterly, or even twice a year, and it has the potential to improve patient compliance [57]. Currently, ALN-HBV has entered the clinical trial stage [58].

### 5.3. Gene Editing Technology

The development of nucleic acid-based therapies for hepatitis B has been severely restricted due to several undesirable side-effects and methodological limitations. With the emergence and development of three artificial endonucleases, namely, zinc finger nuclease (ZFN), transcription activator-like effector nuclease (TALEN), and clustered regularly interspaced short palindromic repeats (CRISPR)-associated protein 9 (CRISPR/Cas9), studies have been conducted on the use of these gene editing tools in the treatment of HBV infection [74,75,76,77].

The nuclease in the zinc finger system combines a zinc finger protein with a specific DNA sequence and specifically cleaves the target DNA by Fok I endonuclease [78]. TALENs are obtained from a special plant pathogen, *Xanthomonas*. It can edit and cut the target DNA by combining the transcription activator-like protein with a specific DNA sequence [79].

CRISPR/Cas9 is a type of RNA-guided Cas endonuclease for specific DNA modification of targeted genes [80]. Compared to ZFNs and TALENs, the CRISPR/Cas9 system has the advantages of convenient construction, low cost, and the ability to edit a wide range of genes. Multiple single-guide RNAs (sgRNAs) for target genes can be designed and edited simultaneously. In addition, the CRISPR/Cas9 system is more accurate and safer than ZFNs and TALENs. Therefore, the CRISPR/Cas9 system has become the most promising technology [81,82,83] and has attracted extensive attention in the field of HBV gene editing [84,85].

Several studies have shown that the CRISPR/Cas9 system can efficiently disrupt the HBV genome [86,87], as well as disrupt or inactivate the cccDNA [88,89,90] and integrated genomes [59,91], which is important for the radical cure of CHB. However, several problems persist in the clinical application of CRISPR/Cas9 technology [92,93], such as limited detection ability, single index, absence of comprehensive studies regarding the effectiveness and duration of effect, repair of HBV DNA, possible off-target effects, and the impact on the host cell genome. Efficient delivery of CRISPR/Cas9 into HBV-infected hepatocytes and limiting the unintended off-target effects are important for the technology to be considered for clinical applications. Adeno-associated virus (AAV)-assisted delivery of the CRISPR/Cas9 system has shown improved gene targeting efficacy in vivo [94,95]. However, prolonged persistence and immunogenicity of AAV in the host prevent the wide therapeutic application of AAV-based CRISPR/Cas9 delivery [96]. Nanoparticle drug delivery technology has the potential, as a nonviral vector, to improve the effectiveness, enhance the targeting ability, and reduce the off-target effects and host gene toxicity of the CRISPR/Cas9 system [97,98,99,100,101]. Surprisingly, recent reports demonstrated some success in using LNPs to deliver CRISPR/Cas9 components [102].

Jiang et al. [59] reported the use of lipid-like nanoparticles (LLNs), which could effectively deliver Cas9 mRNA and sgRNA to the liver and achieve in vivo targeting of HBV DNA.

Suzuki et al. [60] reported an LNP-based CRISPR/Cas9 ribonucleoprotein delivery nanoplatform synthesized using a clinically relevant mixer-equipped microfluidic device. The formulation exhibited excellent gene disruption (up to 97%) and base substitution (up to 23%) without any apparent cytotoxicity.

However, while nonviral delivery technologies for the CRISPR/Cas9 system appear promising, difficulties associated with the synthesis of clinically relevant formulations and the poor efficiency of delivery severely hinder therapeutic genome editing. Therefore, further study is required on nanoparticle-based CRISPR/Cas9 delivery for genome editing and inhibition of HBV.

## 6. Nanoparticle Delivery Systems for Vaccines

Compared with conventional vaccines, nanovaccines have unparalleled advantages [103], which are a size and morphology similar to those of pathogens, the enhancement of antigen presentation, and passive arrival at the epicenter of the immune response. A nanovaccine against HBV was first licensed in 1986, following which nanotechnology has been increasingly used for the development of antiviral vaccines [104,105]. As shown in Table 3, we summarize the composition and properties of nanoparticle delivery systems for anti-HBV vaccines.

### 6.1. Polymeric Nanoparticles

Polymeric nanoparticles for vaccine delivery improve antigen bioavailability in vivo and influence the innate and adaptive immune responses, including the generation of memory cells, which are essential for the efficacy of a vaccine.

Wang et al. [106] designed a ferritin nanoparticle vaccine that can deliver preS1 to specific myeloid cells, including SIGNR1^+^ dendritic cells (which activate T follicular helper cells) and lymphatic sinus-associated SIGNR1^+^ macrophages (which can activate B cells). This nanoparticle vaccine induced a high-level and persistent anti-preS1 response that resulted in efficient viral clearance and partial serological conversion in a CHB mouse model. Thus, the vaccine offers a promising translatable strategy for the functional cure of CHB.

Zhu et al. [107] reported the use of mannose-modified poly (d,l-lactide-*co*-glycolic acid) nanoparticles to load HBsAg (HBsAg/MNPs) as a model antigen for targeted and sustained delivery of hepatitis B vaccine through subcutaneous injection to elicit high-level immunoprophylaxis. Mannose, as a targeted modification, could enhance the uptake of nanoparticles by the immune cells and improve the humoral and cellular immune responses.

### 6.2. Virus-like Particles

Virus-like particles (VLPs), which are morphologically identical to the native infectious virus particles, constitute versatile tools in vaccine development due to their favorable immunological characteristics such as size, repetitive surface geometry, and the ability to induce both innate and adaptive immune responses. Moreover, VLPs are safe templates and are cost-effective.

In the 1980s, the first-generation HBsAg VLP-based vaccine was produced using HBsAg VLPs isolated from the blood of infected individuals; however, it was associated with several issues, including biosafety and tolerability [112]. The second-generation hepatitis B vaccine was based on the expression of a small HBV protein in cell cultures of *Saccharomyces cerevisiae* yeast, which could overcome many drawbacks of the first-generation vaccine [113]. Furthermore, a third-generation HBsAg VLP-based vaccine was developed, licensed as Sci-B-Vac™. This vaccine incorporates three HBV surface antigens (S, preS1, and preS2 antigens) and is expressed in mammalian Chinese hamster ovary cells. Sci-B-Vac™ can induce high titers of anti-HBsAg antibodies in low doses and can additionally induce protective antibodies directed against preS1 and preS2. The vaccine has demonstrated better responses in older and obese individuals, immunocompromised patients, low responders, patients with renal failure, and those who have undergone transplantation or are under dialysis [114].

Although effective prophylactic vaccines against HBV have been available for a long time, they remain ineffective in chronic infection. Moreover, some individuals do not demonstrate positive immune responses after being vaccinated with the commercial hepatitis B vaccines. Therefore, novel VLPs have continuously been exploited as effective entities in HBV vaccine delivery.

In the study by Yong et al. [108], the “a” determinant within the HBsAg was displayed on the recombinant capsid protein of *Macrobrachium rosenbergii* nodavirus, which can be purified easily in a single step through immobilized metal affinity chromatography. The purified protein self-assembled into the VLPs. Immunization of BALB/c mice with this chimeric protein induced specific antibodies against the “a” determinant. In addition, it induced significantly more natural killer and cytotoxic T cells and increased IFN-γ secretion, which are vital for virus clearance.

Ninyio et al. [109] produced self-assembled VLPs of the prawn nodavirus capsid (Nc) displaying the HBV “a” determinant (aD) on *Spodoptera frugiperda* (Sf9) cells (Nc-aD-Sf9). An enzyme-linked immunosorbent assay (ELISA) revealed that the aD epitope of the VLPs was significantly antigenic to anti-HBsAg antibodies. In addition, multiplex ELISA of serum samples from the vaccinated mice showed significant induction of IFN-γ, IL-4, IL-5, IL-6, IL-10, and IL-12p70. This cytokine profile is indicative of natural killer cell, macrophage, dendritic cell, and cytotoxic T-lymphocyte activities, suggesting prophylactic innate and adaptive cellular immune responses mediated by Nc-aD-Sf9 VLPs.

Mobini et al. [110] designed a VLP-based vaccine by placing the antibody-binding fragments of HBsAg in the major immunodominant region epitope of HBcAg to stimulate multilateral immunity, owing to the pivotal role of specific immune responses to HBsAg and HBcAg in infection control. Modeling and molecular dynamics demonstrated the folding stability of HBcAg as a carrier while inserting Myrcludex B and the “a” determinant of HBsAg. The final construct showed promise in inducing humoral and cellular responses against HBV.

The primary cause of CHB and the greatest impediment for a therapeutic vaccine are the direct and indirect effects of immune tolerance to HBV antigens. The resultant defective CD4^+^/CD8^+^ T-cell response, poor cytokine production, insufficient neutralizing antibodies (nAbs), and poor response to HBsAg vaccination characterize CHB infection. Whitacre et al. [111] developed VLPs that elicited nAbs to prevent the spread of the virus and prime CD4^+^/CD8^+^ T cells to eradicate intracellular HBV.

## 7. Discussion

Following several years of research worldwide, there has been significant progress in the understanding of the mechanism and treatment methods for HBV infection, and several novel anti-HBV therapeutics are in preclinical development or early clinical trials. Nanoparticles present a high hepatocyte-targeting efficiency and antiviral effect for the delivery of a diverse array of drugs/materials, including anti-HBV nucleoside drugs, gene therapy, and vaccines.

Several advances have contributed to positioning nanoparticle drug delivery systems at the forefront of the discovery of a functional cure for HBV. First, the novel candidate drugs and nanoparticle drug delivery systems, especially polymeric nanoparticles and lipid nanoparticles, provide hope for improving the inhibition rate of viral replication, especially in the clearance of cccDNA for radical treatment of CHB. Second, on the basis of a comprehensive understanding of the tissue and intracellular environments, several receptor-specific ligands have been embodied in the design of nanocarrier systems, to achieve higher therapeutic efficacy. Third, one of the ideal properties of nanocarriers, which is an advantage for anti-HBV therapy, is the ability to combine the drugs needed for liver imaging.

Despite encouraging results so far, the formulation and improvement of novel targeted drug delivery schemes remain in the early stages, and the following suggestions are proposed for further research on targeted anti-HBV.

(1) Further research on nanoparticle drug delivery systems is required for their effective distribution in vivo, efficient cell targeting, drug enzyme protection, safe and stable delivery, and rapid drug release, among other aspects.
(2) Future anti-HBV strategies should consider combined treatment methods, such as NAs, immune activators, and therapeutic vaccines, in order to significantly improve the quality of life of patients with CHB and achieve the ideal goal of a functional and complete cure.
(3) Researchers should pay attention to the latest progress in the basic research on HBV and anti-HBV treatment, as well as identify and evaluate the factors that affect anti-HBV nanoparticle delivery in vivo, thereby improving its efficiency.

## 8. Conclusions

In this narrative review, the current nanoparticle delivery systems for anti-HBV therapy, including nucleoside drugs, gene therapy, and vaccines, were summarized. Their potential curative significance and the progress toward clinical application were also discussed. Despite several existing challenges, the field is gaining momentum, and significant progress seems imminent.

## Figures and Tables

**Table 1:** Composition and properties of nanoparticle delivery systems for anti-HBV nucleoside drugs.

Nanopreparation	Nanocarrier Composition	Ligand	Drug	Preparation Method	Particle Size (nm)	Encapsulation Efficiency (%)	Drug Loading (%)	Ref.
Polymeric nanoparticle	Stearic acid-grafted chitosan oligosaccharide (grafting rate 3.79%)	N/A	Lamivudine (ester linkage to stearic acid)	Dialysis method	273.8 ± 8.5	99.48 ± 0.04	39.04 ± 0.51	[20]
	Low-molecular-weight chitosan	Glycyrrhizin	Lamivudine	Ionotropic gelation method	145.8 ± 4.2	65.89 ± 1.58	71.37 ± 1.19	[21]
Solid lipid nanoparticle	Monostearin (poloxamer 188 as surfactant)	N/A	Adefovir dipivoxil	Solvent diffusion method	389.4 ± 166.5	15.32 ± 2.58	3.06 ± 0.51	[19]
Multiple lipid nanoparticle	Compritol^®^ ATO 888, Miglyol^®^-812 (Span-80 as surfactant)	N/A	Lamivudine	Hot homogenization method combined with high shear and ultrasonication	450 ± 10	20 ± 2	1.08 ± 0.06	[22]

**Table 2:** Composition and properties of nanoparticle delivery systems for anti-HBV genetic drugs.

Nanopreparation	Nanocarrier Composition	Ligand	Drugs	Method of Preparation	Particle Size (nm)	Encapsulation Efficiency (%)	Drug Loading (%)	Refs.
Ribozyme technology								
Polymeric micelle	Chitosan oligosaccharide-grafted stearic acid	N/A	10–23 DNAzyme specific to e-gene ORF A^1816^UG	Self-aggregation	164.0 ± 2.1	N/A	N/A	[47]
	Chitosan oligosaccharide-SS-Octadecylamine	N/A	10–23 DNAzyme specific to e-gene ORF A^1816^UG	Self-aggregation	214.75 ± 3.43	96.48 ± 0.27	1.582 ± 0.004	[48]
			10–23 DNAzyme specific to s-gene ORF A^157^UG		230.70 ± 6.16	96.45 ± 0.33	1.581 ± 0.005	
RNA interference technology								
Lipid nanoparticle	Cationic cholesteryl polyamine *N*1-cholesteryloxycarbonyl-3,7-diazanonane-1,9-diamine and the neutral colipid dioleoyl-l,r-phosphatidyl ethanolamine	Polyethylene glycol	siRNA	Film dispersion– sonication method	80–100	N/A	N/A	[49]
	Proprietary lipid nanoparticle platform (Arbutus Biopharma)	N/A	ARB-1740	Spontaneous vesicle formation	65–80	92–98	N/A	[50,51]
			ARB-1467		N/A	N/A	N/A	[52]
Polymeric nanoparticle	Poly(d,l-lactide-*co*-glycolide)-grafted chitosan (PLGA–CHS)	N/A	Plasmid DNA (pDNA)	Spontaneous emulsion diffusion method	59.43 ± 14	N/A	Nearly 100% at the ratio of 100:1 (PLGA–CHS NS to pDNA)	[53]
	ARC-EX1 containing hepatocyte-targeted *N*-acetylgalactosamine-conjugated melittin-like peptide	N/A	ARC-520 along with a related ARC-521	N/A	N/A	N/A	N/A	[54,55]
Conjugate nanoparticle	*N*-acetylgalactosamine (GalNAc)–siRNA conjugates	N/A	ALN-HBV	N/A	N/A	N/A	N/A	[56,57,58]
Gene editing technology								
Lipid-like nanoparticle	Tris(2-aminoethyl) benzene-1,3,5-tricarboxamide	N/A	CRISPR/Cas9	N/A	N/A	N/A	N/A	[59]
Lipid nanoparticle	Cationic lipid, phospholipid, cholesterol	Polyethylene glycol	CRISPR/Cas ribonucleoprotein	Mixer-equipped microfluidic device	<200	>80	N/A	[60]

**Table 3:** Composition and properties of nanoparticle delivery systems for anti-HBV vaccines.

Nanopreparation	Nanocarrier Composition	Vaccine	Method of Preparation	Particle Size (nm)	Encapsulation Efficiency (%)	Drug Loading Capability	Ref.
Polymeric nanoparticle	SpyTag–ferritin	PreS1	Self-assembly	N/A	N/A	N/A	[106]
	Mannose-modified poly d,l-lactide-*co*-glycolic acid	HBsAg	Double emulsion solvent evaporation technique	186.6 ± 3.7	63.7 ± 4.5	1.5 ± 0.1 μg/mg	[107]
Virus-like particles (VLPs)	Macrobrachium rosenbergii nodavirus (MrNV)	MrNV VLPs	Self-assembly	30	N/A	N/A	[108]
	HBV “a” determinant (aD) displayed on the prawn nodavirus capsid (Nc) and expressed in *Spodoptera frugiperda* (Sf9) cells (Nc-aD-Sf9)	Nc-aD-Sf9 VLPs	Self-assembly	56.4	N/A	N/A	[109]
	HBcAg including Myrcludex and the “a” determinant sequence of HBsAg	VLP-based vaccine by placing the antibody-binding fragments of HBsAg in the major immunodominant region epitope of HBcAg	Self-assembly	N/A	N/A	N/A	[110]
	The woodchuck hepatitis core antigen (WHcAg)	PreS1-WHc VLPs	Self-assembly	N/A	N/A	N/A	[111]

## Data Availability

Not applicable.
